# Guided by light, microtubules choreograph the directional expansion of cotyledons

**DOI:** 10.1093/plphys/kiaf234

**Published:** 2025-06-06

**Authors:** Blanca Jazmin Reyes-Hernández

**Affiliations:** Assistant Features Editor, Plant Physiology, American Society of Plant Biologists; Faculty of Science, Department of Plant and Environmental Sciences, Section for Plant Glycobiology, University of Copenhagen, Frederiksberg 1871, Denmark

In 1880, Charles Darwin and his son Francis observed one of the first clues that light can direct plant growth, describing how young plants sense light at their tip, causing the stem to bend toward the light. Today, we know that light not only controls stem bending but also shapes the size and form of leaves and shoots. For example, seedlings exposed to light open their small embryonic leaves (cotyledons), making them broader and rounder, whereas in darkness, cotyledons remain small and elongated in an oval shape. Although many studies have advanced our understanding of light signaling, the cellular mechanisms by which light triggers these morphological changes are still not fully understood.

Plants possess specialized photoreceptors to detect different types of light. Among them, the phytochromes absorb wavelengths between approximately 600 and 750 nm, classified as red (600 to 700 nm) and far-red (700 to 750 nm) light. Arabidopsis has 5 phytochromes (phyA to phyE) with overlapping roles in controlling plant development. Phytochromes act as molecular switches, cycling between an “off” form (Pr), which absorbs red light, and an “on” form (Pfr), which absorbs far-red light. Red light converts Pr into Pfr (photoactivation), while far-red light or darkness reverts Pfr back to Pr (Legris et al. 2019). Upon photoactivation, Pfr translocates from the cytosol to the nucleus, where it binds transcription factors such as PHYTOCHROME-INTERACTING FACTORS (PIFs), promoting their degradation and releasing the repression of light-responsive genes (Chen and Chory 2011).

Once seedlings perceive light, phytochrome signaling rapidly modifies cell expansion and organ development. In darkness, the embryonic stem (hypocotyl) elongates, while in the light, the hypocotyl remains short. At the cellular level, a key player in hypocotyl elongation is the array of cortical microtubules (CMTs) (Krahmer and Fankhauser 2024), tubular protein filaments made of tubulin located beneath the plasma membrane. CMTs guide the deposition of cellulose microfibrils into the cell wall, determining the direction of expansion. In dark-grown hypocotyls, microtubules are arranged transversely to the growth axis, reinforcing lateral walls and promoting longitudinal elongation (Krahmer and Fankhauser 2024). Light exposure reorganizes CMTs into a more longitudinal orientation, restricting hypocotyl elongation and promoting lateral expansion (Lian et al. 2017).

Similar to hypocotyls, phytochromes stimulate cotyledon expansion in response to light. In darkness, cotyledons remain small and oval, but exposure to light makes them larger and rounder. In Arabidopsis, phyB in its “on” form plays a central role in this process (Neff and Van Volkenburgh 1994). Although hormonal and cytoskeletal pathways regulate polar expansion (growth more along one axis than another) in true leaves (Romanowski et al. 2021), it was unclear whether similar mechanisms underpin cotyledon polar expansion.

A recent study published in *Plant Physiology* (Cho and Choi 2025) explores the cellular basis of phytochrome-mediated changes in cotyledon shape. Using *phyA* and *phyB* mutants, the authors measured cotyledon length and width under red and far-red light. By calculating the length-to-width ratio (leaf index), they showed that phyA promotes polar expansion under far-red light, while phyB promotes similar growth under red light. Each mutant remained blind to their respective activating light wavelengths, displaying small, oval cotyledons similar to those grown in darkness.

To understand the cellular behavior behind cotyledon polar expansion, the authors examined epidermal pavement cells using confocal microscopy. This provided information on whether polar growth results from polar cell proliferation, polar cell expansion, or both. Their results indicated that polar growth primarily results from reduced longitudinal growth (length) compared to increased lateral growth (width) and not from enhanced cell proliferation in one preferred direction. PhyA and phyB are necessary for this light-driven cell remodeling, with phyA acting under far-red light and phyB under red light.

Because PIFs, which act downstream of phytochromes, suppress light responses, the authors analyzed their role in cotyledon polar expansion. Studying the *pif1/3/4/5* quadruple mutant (*pifq*) and PIF4-overexpression plants, they found that PIFs inhibit cotyledon and cell expansion. In darkness, loss of PIFs resulted in larger, rounder cotyledons (resembling those under red light), whereas PIF4 overexpression produced more elongated cotyledons even under red light. These findings suggest that PIFs, while generally limiting expansion, preferentially restrain transverse (width) growth, leading to elongated, oval-shape forms.

In hypocotyls, PIFs promote elongation via auxin biosynthesis and signaling. To test auxin's role in polar expansion, Cho and Choi treated plants with picloram (a synthetic auxin). Picloram reduced cotyledon size and slightly induced an oval shape under red light (resembling dark-grown morphology), while the *axr3* mutant (which impairs auxin signaling) developed larger, rounder cotyledons in the dark. Their results indicate that auxin partially mediates the inhibitory role of PIFs on polar expansion in cotyledons.

The authors next identified *LONGIFOLIA* genes (*LNG1* and *LNG2*) as potential downstream regulators of the phyB-PIF module. Interestingly, LNG1 and LNG2 encode microtubule-associated proteins and were previously reported to regulate leaf shape (Lee et al. 2006). Expression analyses (reverse transcription quantitative-PCR) confirmed that red light reduced *LNG1* and *LNG2* transcript levels via PIF inhibition. Mutants lacking all LNGs (*lng1/2/3/4*) showed a rounder shape in darkness, while LNG1 overexpression promoted pronounced polar expansion along the length of cells and cotyledons, confirming that LNGs promote elongation downstream of the phyB-PIF module. To further explore LNG function, the authors examined microtubule organization using GFP-Tubulin lines. In darkness, CMTs were predominantly arranged transversely to the main growth axis, while light triggered a shift toward longitudinal orientations. This rearrangement was disrupted in *phyB*, *pifq*, and *lng1/2/3/4* mutants, indicating that light controls microtubule patterns through the phyB-PIF-LNG pathway, guiding the direction of cotyledon expansion.

This work provides new insights into how light sculpts the embryonic leaves of plants after germination by linking phytochrome activation and microtubule organization to promote growth directionality (Figure). Nonetheless, open questions remain; for example, what is the mechanism by which the LNG promote transverse microtubule arrangements? It also remains to be determined whether similar mechanisms operate in true leaves or whether cotyledon-specific programs are involved. Additionally, whether these findings extend to monocots, which have a single cotyledon that typically does not expand and round out like in Arabidopsis, remains unknown. For example, characterizing rice LNG homologs, which is currently limited, and determining whether PIFs also regulate the shape of monocots cotyledons will be an exciting avenue for future research.

**Figure.**
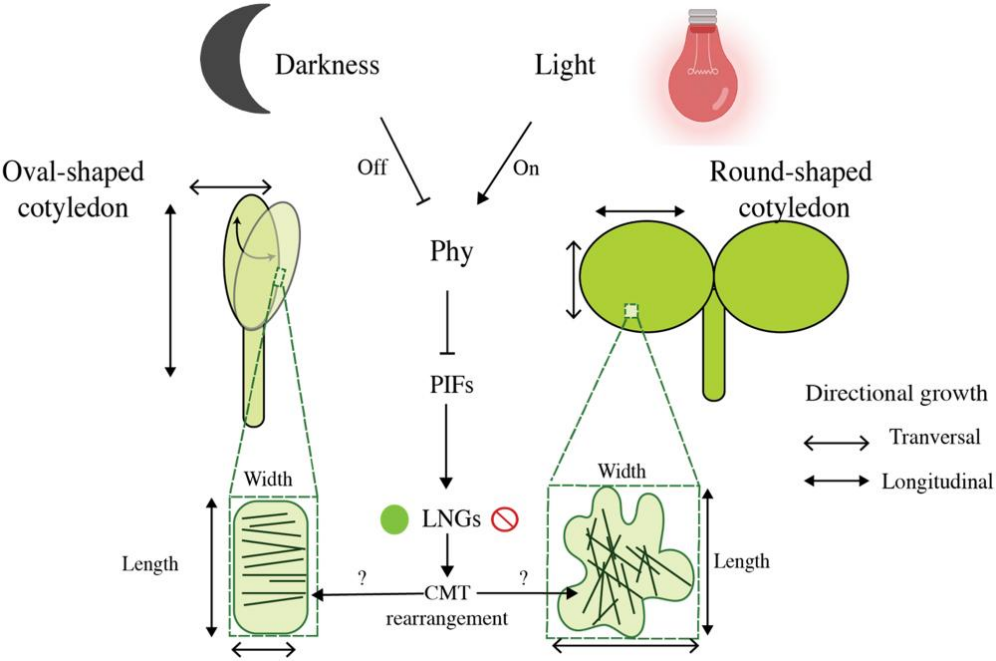
Working model of cotyledon and pavement cell expansion via the phyB–PIF–LNG pathway. In dark-grown seedlings, cotyledon pavement cells (illustrated within the dashed rectangle) display transversely arranged CMTs (represented by dark green lines within the cells) promoted by high expression of the PIF target genes, LNGs (filled circle). This arrangement favors longitudinal expansion (doubleheaded arrow). However, PIFs also inhibit overall growth in the dark, resulting in small, elongated pavement cells and small, oval-shaped cotyledons (left cotyledon representation). Upon red light exposure (right cotyledon representation), phyB inhibits PIF activity, allowing pavement cells to expand proportionally along both longitudinal and transverse directions. Light reduces transverse CMTs by downregulating LNGs (red crossed-out circle). This shift weakens longitudinal relative to transverse growth (double-headed open arrow), favoring the formation of round-shaped cotyledons under light conditions. PIFs and LNGs are unlikely to be the sole regulators of cotyledon polar expansion; other factors may influence LNG function or CMT rearrangement (question mark).

## Data Availability

No additional data were produced or assessed to support this research.
